# Adenocarcinoma of the Small Bowel: A Surgical Dilemma

**DOI:** 10.4103/1319-3767.56105

**Published:** 2009-10

**Authors:** Ketan Vagholkar, Tony Mathew

**Affiliations:** Department of Surgery, Padmashree Dr. D. Y. Patil Medical College and Rajawadi Municipal General Hospital, Ghatkopar, Mumbai - 400 077, India

**Keywords:** Small intestine, cancer, tumor, adenocarcinoma

## Abstract

Malignant tumors of the small intestine are among the rarest types of gastrointestinal cancers. Due to their infrequent occurrence and the multitude of tumor types (viz, adenocarcinomas, carcinoids, sarcomas, and lymphomas), not much is known about their natural history and presentation, and there is often delay in the diagnosis. Adenocarcinoma is the commonest histologic type of small bowel cancer. There are no prospective randomized trials that have elucidated the best diagnostic and therapeutic options for this rare condition. In this article, a case of adenocarcinoma of the jejunum presenting as an abdominal lump is presented, along with a review of the literature.

Adenocarcinoma of the small bowel is a rare malignancy as compared to other malignancies of the gastrointestinal tract. However, it is the commonest amongst small bowel cancers. It is asymptomatic in the early stages and by the time it becomes symptomatic it has usually spread to other sites. Thus, the diagnosis is often delayed, with consequent poor prognosis.

## CASE REPORT

A 47-year-old male presented with the complaint of an abdominal mass in the right lumbar region. He was admitted for evaluation. He did not have symptoms suggestive of intestinal obstruction and there had been no weight loss. There was no history of hematemesis or melena. Physical examination revealed an intra-abdominal mass that did not move with respiration. The mass was not ballottable.

**Figure 1:**
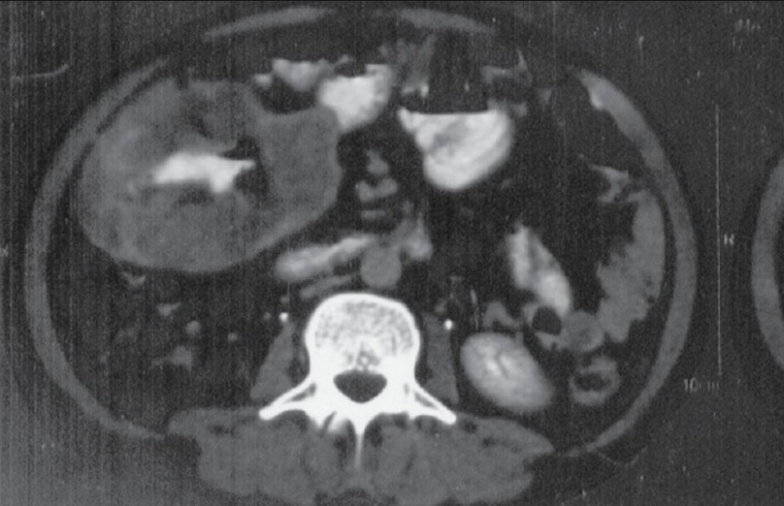
CT scan showing the lesion involving the small intestine

**Figure 2:**
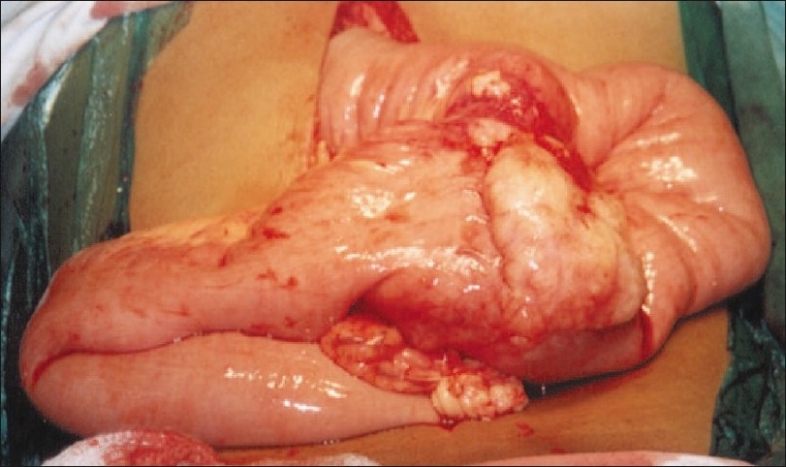
Solid concentric lesion involving the jejunum

**Figure 3:**
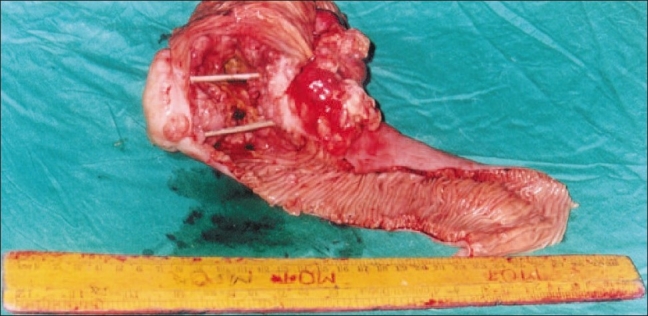
Resected specimen which has been cut open to show the mass

**Figure 4:**
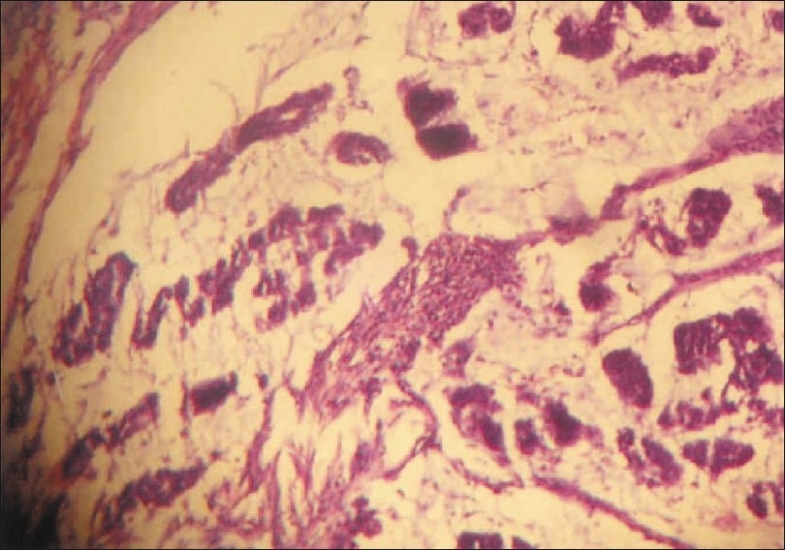
Photomicrograph showing tumor cells arranged in irregular acini (H and E, ×400)

CT scan revealed a circumferential growth involving the small bowel [Figure 1]. There were no hepatic metastases nor was there any free fluid in the peritoneal cavity. The patient underwent an exploratory laparotomy, at which the mass was seen encircling the jejunum [Figure 2]. The involved segment was resected [Figure 3]. Postoperative recovery was uneventful. Histopathology of the specimen revealed adenocarcinoma of the small intestine [Figure 4]. The resection margins were clear of tumor cells on histology and there was no evidence of lymph node involvement. The lesion was T3N0M0, i.e., stage II disease. Adjuvant chemotherapy was not given as the patient did not have metastases and the margins were clear. The patient has been followed up for 2 years and there has been no evidence of recurrence.

## DISCUSSION

The small intestine constitutes about 90% of the mucosal surface area and 75% of the length of the gastrointestinal tract. Despite this, malignant neoplasms are uncommon in the small intestine, constituting only 20% of all the gut tumors. Many theories have been postulated to explain the low incidence of malignant tumors at this site. For example, it has been said that the large volumes of alkaline fluid in the small bowel, the presence of various enzymes, and the high immunoglobulin A levels cause dilution and detoxification of potential carcinogens and also prevent prolonged contact of such carcinogens with the mucosa. In addition, the small intestine has a very limited number of bacteria (as compared to the colon) that are capable of transforming potential procarcinogens into their active breakdown products. Since the incidence of small bowel carcinoma is very low, not much information is available regarding the molecular aspects of these tumors, which could be of help in the planning, prevention, diagnosis, and management of these tumors.

In general, small intestinal cancers have a low prevalence in Asian countries as compared to the West. Males have a higher predilection for these malignancies. Increasing age is associated with a higher incidence of small intestinal cancers.

Genetic factors have been strongly implicated in the etiology of adenocarcinoma of the small intestine. Patients suffering from familial adenomatous polyposis have a higher chance of developing duodenal adenocarcinoma.[1] These patients have high frequency of p53 overexpression.[2] Hereditary nonpolyposis colorectal cancer patients have a high likelihood of developing adenocarcinoma of the small bowel.[3] Environmental factors such as a diet rich in red meat, salt-cured or smoked foods, as well as intake of tobacco and alcohol, have been implicated in the etiology of this malignancy.[4]

Predisposing medical conditions are Crohn disease and celiac disease (nontropical sprue). Patients suffering from Crohn disease have a high risk of developing adenocarcinoma of the small bowel, whereas patients suffering from celiac disease have increased risk of developing small bowel lymphoma rather than adenocarcinoma.[5]

Approximately 64% of all small bowel tumors are malignant and 40% of the tumors are adenocarcinomas.[6] The remaining malignancies comprise GIST (gastrointestinal stromal tumors), carcinoids, and lymphomas).[7] These tumors have a resemblance to large bowel adenocarcinomas. Adenocarcinomas in the small bowel arise from premalignant adenomas. Through a stepwise accumulation of genetic mutations, these adenomas become dysplastic and progress to carcinoma *in situ* and then to invasive adenocarcinomas. They metastasize via the lymphatics or portal circulation to the liver, lung, bone, brain, and other distant sites. Small bowel adenocarcinomas tend to cluster away from the colon, toward the gastric end of the small intestine.[8] Approximately 50% arise in the duodenum, 30% in the jejunum, and 20% in the ileum. *K-ras* mutation and p53 overexpression are common in small bowel cancers.[2] Impaired small intestinal acyl coA thioesterase synthesis is probably related to the adenoma-carcinoma sequence of small intestinal epithelial tumors.[9] A significant number of small bowel tumors show moderate to strong COX-2 and c PLA 2 expression.[10]

Small bowel cancers are asymptomatic in the early stages. As the disease progresses, symptoms develop. The nature of symptoms is nonspecific and, as a result, there is a delay in diagnosis which averages 6-8 months.[11] Abdominal pain and weight loss are the commonest symptoms. Bleeding, vomiting, nausea, and obstruction are less common. Physical examination in the early stages is unremarkable. Rarely, a palpable abdominal mass or a tender abdomen, possibly with peritoneal signs, may be found due to obstruction or perforation.

Laboratory tests may show mild anemia due to chronic blood loss. Liver function test may reveal hyperbilirubinemia in case of duodenal tumors. Elevated transaminases may be found in case of liver metastasis. The diagnosis of small bowel adenocarcinomas may be elusive. Upper GI series, with small-bowel follow-through, shows abnormalities in 53.83% of patients with small bowel cancers.[12] Abdominal CT scan will reveal the exact site and extent of local disease as well as the presence of liver metastasis.[13] Upper GI endoscopy with small-bowel enteroscopy may allow identification and biopsy of lesions in the duodenum and jejunum. Videocapsule endoscopy has shown promise in the diagnosis of small bowel disorders.[14,15]

Surgical resection provides the only hope for cure for adenocarcinoma of the small bowel.[16,17] Patients with a lesion in the proximal duodenum should undergo pancreaticoduodenectomy.[17,18] For lesions elsewhere in the small bowel, the treatment is wide resection. Surgery, in the form of a bypass for intestinal obstruction, may also be indicated for palliation in patients with symptomatic advanced disease.[17] Chemotherapy is of benefit as an adjuvant to surgery, especially when there is metastatic disease. Jigyasu *et al.* studied 14 subjects with metastatic small bowel adenocarcinoma treated with chemotherapy regimens, mostly containing 5-fluorouracil (5-FU). Two minor responses and one partial response occurred, with a median survival of 9 months.[19] Ouriel and Adams reported a mean survival of 10.7 months in 6 patients with metastatic disease treated with 5-FU-based regimens, compared with a mean survival of 4 months in 6 patients with metastatic disease who received no chemotherapy.[20] Crawley *et al*. reported eight patients with advanced small bowel adenocarcinoma treated with infusional 5-FU- based regimens and found a response rate of 37.5% and a median survival of 13 months.[21] Polyzos *et al*. reported minor responses, with improvement of symptoms, using salvage irinotecan therapy for 5-FU-refractory small bowel adenocarcinoma.[22] Bettim *et al*. found that the FOLFOX-4 regimen (which is a combination of infusional 5-FU, oxaliplatin, and leucovorin) could be safely administered as adjuvant chemotherapy in three subjects with resected small bowel adenocarcinoma associated with celiac disease.[23]

Veyrieves *et al*. reported an overall 5-year survival of 38%; with palliative treatment alone the 5-year survival was 0%, while it was 54% after curative resection. In patients undergoing curative resection, the 5-year survival was 63% when lymph nodes were not involved and 52% when they were, 57% when the serosa was not involved and 53% when it was, 56% when the tumor was well or moderately well differentiated and 40% when it was undifferentiated. Other factors influencing long-term survival were the emergency presentation, the site, the multiplicity, and the size of the tumor.[24]

In conclusion, primary small bowel tumors are rare and the prognosis is generally considered to be poor. Diagnosis is often difficult because of the infrequency of these tumors and the nonspecific symptoms. Aggressive surgical resection in an attempt to achieve complete tumor removal should be the aim. Large tumor size, advanced histological grade, and transmural invasion are associated with decreased survival.[25]
